# Three Strains of *Lactobacillus* Derived from Piglets Alleviated Intestinal Oxidative Stress Induced by Diquat through Extracellular Vesicles

**DOI:** 10.3390/nu15194198

**Published:** 2023-09-28

**Authors:** Shengkai Feng, Yihan Liu, Jing Xu, Jinping Fan, Jingjing Li, Zhifeng Wu, Yue Sun, Wen Xiong

**Affiliations:** College of Animal Sciences and Technology & College of Veterinary Medicine, Huazhong Agricultural University, Wuhan 430070, China; fengsk@webmail.hzau.edu.cn (S.F.); liuyinhan@webmail.hzau.edu.cn (Y.L.); 15953689552@163.com (J.X.); jinpingfan@webmail.hzau.edu.cn (J.F.); lijingjing@webmail.hzau.edu.cn (J.L.); wuzf@webmail.hzau.edu.cn (Z.W.); 18293386804@163.com (Y.S.)

**Keywords:** *Lactobacillus*, gut microbiota, oxidative stress, extracellular vesicles, *Poria cocos* polysaccharide

## Abstract

Previous studies found that *Poria cocos* polysaccharides (PCPs) significantly enhanced the antioxidant activity in piglet intestines while increasing the abundance of *Lactobacillus*. However, the relationship between *Lactobacillus* and antioxidant activity has yet to be verified, and the mode of action needs further investigation. Six *Lactobacillus* strains isolated from the intestines of neonatal piglets fed with PCPs were studied to investigate the relationship between *Lactobacillus* and intestinal oxidative stress. The results showed that three of them alleviated intestinal oxidative stress and protected the intestinal barrier. Subsequently, we extracted the extracellular vesicles (EVs) of these three *Lactobacillus* strains to verify their intestinal protection mode of action. We found that these EVs exerted an excellent antioxidant effect and intestinal barrier protection and could directly improve intestinal microbial composition. Our findings suggested that the EVs of the three *Lactobacillus* strains could enhance antioxidant activity by improving the physical intestinal barrier and remodeling gut microbiota. Unlike probiotics, which should be pre-colonized, EVs can act directly on the intestines. This study provides new ideas for the subsequent development of products to protect intestinal health.

## 1. Introduction

*Poria cocos* is the dry, edible, large sclerotium of *Wolfiporia cocos* (F. A. Wolf), known as Fu Ling in China, Hoelen in Japan, and tuckahoes or Indian bread in North America. It is a famous traditional Chinese medicine widely used in East Asian countries such as China, Japan, and South Korea. As the traditional Chinese medicine with the strongest “water-inducing” effect, it is commonly used to treat various symptoms, including tissue edema, loss of appetite, palpitations, diarrhea, mental anxiety, insomnia, etc. [1]. The most important active ingredients in the Fu Ling are *Poria cocos* polysaccharides (PCPs), which account for 70–90% of its total weight. Several studies have shown that PCPs have good immunomodulatory, antitumor, anti-inflammatory, and antioxidant effects [2,3,4,5,6,7,8,9,10,11,12]. Based on its multiple beneficial health effects, it often appears on the market as a food or food additive. However, the specific mechanism of action of PCPs has not been fully clarified. Since most PCPs are insoluble, they cannot enter the body directly through the intestines; therefore, studying the relationship between PCPs and gut microbiota is a good starting point [8]. Many previous studies have found that PCPs can improve the diversity and abundance of gut microbiota, suggesting that they participate in gut microbiota regulation [3,12,13].

In our previous study, we found that PCPs significantly increased the antioxidant capacity of the piglet intestines and significantly increased the abundance of six *Lactobacillus* species: *Lactobacillus. delbrueckii subsp. bulgaricus* (*L. delbrueckii*), *Lactobacillus amylovorus* (*L. amylovorus*), *Ligilactobacillus salivarius* (*L. salivarius*), *Lactobacillus acidophilus* (*L. acidophilus*), *Limosilactobacillus fermentum* (*L. fermentum*), and *Ligilactobacillus animalis* (*L. animalis*). As a recognized probiotic, *Lactobacillus* has various beneficial biological roles, such as reducing the incidence of associated metabolic diseases, regulating blood pressure, and exerting anti-inflammatory and antioxidant effects [14]. Based on these findings, we supposed it would be worthwhile to investigate the pathway through which *Lactobacillus* from piglets exerts its antioxidant effect.

Bacterial extracellular vesicles (EVs) participate in a wide range of pathophysiological functions that involve intercellular interactions, including nutrient acquisition, virulence factor delivery, and immune regulation [15]. Recent studies have found that EVs play an important regulatory role in gut microbial homeostasis. Bacterial EVs can trigger a series of immune responses, crucial for gut microbial regulation. For example, the intestinal commensal bacterium can overcome the intestinal epithelium mucus layer barrier by producing EVs and delivering relevant substances to participate in the intestinal immune response, thus preventing colitis in mice [16]. It has also been reported that vesicles isolated from *Escherichia coli* can activate the NOD1 signaling pathway in intestinal epithelial cells. This suggests that extracellular vesicles released by the intestinal microbiota can participate in the immune response and regulate the gut microbiota balance [17]. Other studies have found that *Lactobacillus* EVs in a mouse model of inflammatory bowel disease reduced inflammatory cytokines, decreased serum peroxidase, reduced transmural leukocyte infiltration, lessened the loss of cupped cells in the colon, and ultimately modulated the inflammatory response [18]. EV release usually indicates that the bacteria are metabolically active. Therefore, EVs might reflect the host microbiome activity better than the microbiome itself. EVs might also be an important pathway for the host microbiome to exert its effects. All of this supports the speculation that *Lactobacillus* of piglet origin could act as an antioxidant through EVs.

This study focused on the EVs of *Lactobacillus* species isolated from piglets fed PCPs to study their antioxidant activity, explore how *Lactobacillus* exerts its probiotic effects, and provide new ideas for developing products beneficial to human intestinal health.

## 2. Materials and Methods

### 2.1. Chemical

Diquat (average molecular weight: 344.05) was purchased from Shanghai Fusheng Biotechnology Co., LTD. (Shanghai, China).

### 2.2. Bacterial Isolation and Culture

*L. delbrueckii*, *L. amylovorus*, *L. salivarius*, *L. acidophilus*, *L. fermentum*, and *L. animalis* were isolated from fecal samples of newborn piglets fed PCPs. The frozen *Lactobacillus* culture was inoculated, activated, and passaged three times in solid MRS. Subsequently, the activated bacteria were inoculated into a liquid MRS medium, incubated in an anaerobic incubator at 37 °C for 24 h, centrifuged at 8000× *g* for 5 min, collected, and resuspended in sterile PBS for subsequent experiments.

### 2.3. Bacterial EV Isolation

*L. delbrueckii*, *L. amylovorus*, and *L. salivarius* were incubated in 15 mL of sterile anaerobic MRS broth at 37 °C, and the EVs were isolated from the supernatant. Briefly, 40 mL of logarithmically growing bacterial broth was centrifuged at 8000× *g* for 30 min. The supernatant was collected and centrifuged at 20,000× *g* for 45 min. The resulting supernatant was passed through a 0.22-μm filter (Millipore, Billerica, MA, USA) and centrifuged at 120,000× *g* for 2 h at 4 °C in an sw32 Ti rotor (Beckman Coulter, Fullerton, CA, USA). The pellet was resuspended PBS and centrifuged at 120,000× *g* for 2 h. The supernatant was discarded, and the precipitate was resuspended in 200 μL of sterile PBS for further study. The bacterial EV particle size range was determined using nanoparticle tracking analysis (NTA).

### 2.4. Animal Experiments

C57BL/6J mice were obtained from the Experimental Animal Center of Huazhong Agricultural University (Wuhan, China) after a one-week acclimation period prior to treatment. Mice were kept at 22–25 °C and 12 h light/dark cycles and were allowed to eat and drink freely. All animal experimental and sample collection procedures were approved by the Institutional Animal Care and Use Committee of Huazhong Agricultural University (Hubei, China). All experimental methods followed the Health Guidelines for the Care and Use of Laboratory Animals at Huazhong Agricultural University.

In the *Lactobacillus* gavage experiment, 48 four-week-old C57BL/6J male mice were randomly divided into eight groups (*n* = 6/group); the CON group received 200 µL PBS orally daily and 200 µL PBS intraperitoneally on day 14; the diquat group received 200 µL PBS orally daily and 25 mg/kg BW diquat dissolved in 200 µL PBS intraperitoneally on day 14; and the *L. delbrueckii*, *L. amylovorus*, *L. salivarius*, *L. acidophilus*, *L. fermentum*, and *L. animalis* groups received 200 µL bacterial solution (10^10^ CFU) daily and 200 µL diquat intraperitoneally on day 14. Weight data were recorded daily, and samples (serum, jejunal tissue, and feces) were collected on day 15. The collected fecal samples were frozen immediately in liquid nitrogen and stored at −80 °C.

In the *Lactobacillus* EV gavage experiment, 30 six-week-old C57BL/6J male mice were randomly divided into five groups (*n* = 6/group); the CON group received 200 µL PBS orally daily and 200 µL PBS intraperitoneally on day 0; the diquat group received 200 µL PBS orally daily and 25 mg/kg BW diquat dissolved in 200 µL PBS intraperitoneally on day 0; and the *L. delbrueckii*, *L. amylovorus*, and *L. salivarius* EV groups received 200 µL of bacterial EVs orally daily and 25 mg/kg BW diquat dissolved in 200 µL PBS intraperitoneally on day 0. Body weight data were recorded daily, and samples (serum, jejunal tissue, and feces) were collected on day 5, frozen immediately in liquid nitrogen, and stored at −80 °C.

### 2.5. Morphological Analysis

Jejunum tissue was fixed in 4% formaldehyde, embedded in paraffin, sliced into 5-μm thick slices, and stained with hematoxylin and eosin (H&E). Digital images were captured using light microscopy. The jejunal villus height and crypt depth were measured using CaseViewer (Version 2022.2).

### 2.6. Oxidation Markers’ Assessment

Catalase (CAT), mouse superoxide dismutase (SOD), total antioxidant capacity (T-AOC), glutathione peroxidase (GSH-Px), and malondialdehyde (MDA) were assessed using kits purchased from Shanghai Enzyme Linked Biotechnology Co., LTD. (Shanghai, China).

### 2.7. 16S rRNA Sequencing

Total DNA (200 ng) was extracted from the stool samples using the Rapid DNA Stool Mini Kit (Qiagen Ltd., Hilden, Germany) following the kit instructions. The V3-V4 region was amplified using the 341F-806R primer. The amplified product was detected via agarose gel electrophoresis (2% agarose), recovered with the Axyprep DNA Gel Recovery Kit (Axygen Biosciences, Tewksbury, MA, USA), quantified using a Qubit 2.0 fluorometer (Thermo Fisher Scientific, Waltham, MA, USA), and aggregated into equimolar amounts. Amplicon libraries were sequenced on the Illumina Miseq 2500 platform (Illumina, San Diego, CA, USA) for 250 bp paired-end reads. The 16S raw sequencing reads were demultiplexed based on sample-specific barcodes (6–8 nucleic acids) and imported into the Qiime2 platform (version 2020.2). Amplicon sequence variants were generated using default parameters for quality control and denoising. Phylogenetic trees were generated using default parameters for the Silva132 database. To avoid bias due to different sequencing depths, all samples were refined to 26,527 sequences, with an average superiority coverage of 99.74%.

### 2.8. Statistical Analysis

One-way analysis of variance (ANOVA) and post hoc LSD tests were performed via SPSS 19.0 Statistics (IBM, Armonk, NY, USA). The results are expressed as mean ± SEM. *p* < 0.05 was considered statistically significant. Differences in Alpha diversity were calculated using the Wilcoxon test. Principal coordinate analysis (PCoA) was performed based on the Bray–Curtis and weighted UniFrac distance metrics. Alpha and Beta diversities were calculated using the Vegan package in R (version 4.2.1). Spearman’s correlation analysis was performed for phylum-level bacterial abundance, and *p* < 0.05 was considered statistically significant.

## 3. Results

### 3.1. Lactobacillus Pre-Treatment Attenuates Diquat-Induced Weight Loss and Jejunal Damage

**Figure 1:**
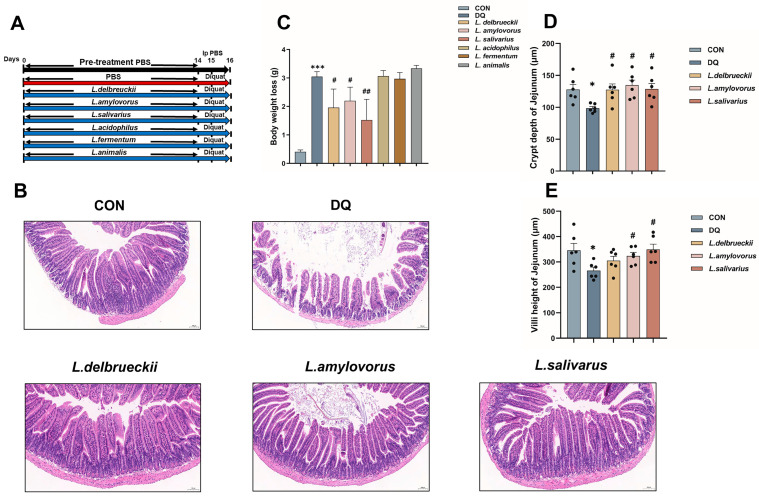
Effect of *Lactobacillus* on weight loss and oxidative damage caused by diquat. (**A**) Experimental grouping scheme. (**B**) H&E staining of the jejunum. (**C**) Mice weight change (*n* = 6). (**D**) Jejunal crypt depth. (**E**) Jejunal villi height. Data are presented as mean ± SEM. When compared to the CON group, * *p* < 0.05; *** *p* < 0.001. When compared to the diquat group, # *p* < 0.05; ## *p* < 0.01. PBS, phosphate-buffered saline; CON, control; DQ, diquat; *L. delbrueckii*, *Lactobacillus delbrueckii subsp. bulgaricus*; *L. amylovorus*, *Lactobacillus amylovorus*; *L. salivarius*, *Ligilactobacillus salivarius*; *L. acidophilus*, *Lactobacillus acidophilus*; *L. fermentum*, *Limosilactobacillus fermentum*; *L. animalis*, *Ligilactobacillus animalis*.

Mice were pretreated for 14 days with six *Lactobacillus* species isolated from the intestines of neonatal piglets gavaged with PCPs. The treatment outcomes are shown in Figure 1A. Compared to CON, the diquat group showed significant weight loss (*p* < 0.001). Gavage of *L. delbrueckii* (*p* < 0.05), *L. amylovorus* (*p* < 0.05), and *L. salivarius* (*p* < 0.01) significantly alleviated the weight loss caused by diquat, whereas gavage of *L. acidophilus*, *L. fermentum*, and *L. animalis* did not. Therefore, we selected the above three groups for the subsequent experiments (Figure 1C).

H&E staining revealed that *L. delbrueckii*, *L. amylovorus*, and *L. salivarius* significantly decreased the damage to the small intestinal epithelium caused by diquat by increasing the number, density, and neat arrangement of the small intestinal microvilli (Figure 1B). Further studies found that *L. delbrueckii*, *L. amylovorus*, and *L. salivarius* significantly increased the jejunal crypts’ depth (Figure 1D; *p* < 0.05), and *L. amylovorus* and *L. salivarius* significantly increased the jejunal villi height (Figure 1E; *p* < 0.05).

### 3.2. Lactobacillus Down-Regulates Serum and Jejunal Levels of Oxidative Indicators

**Figure 2:**
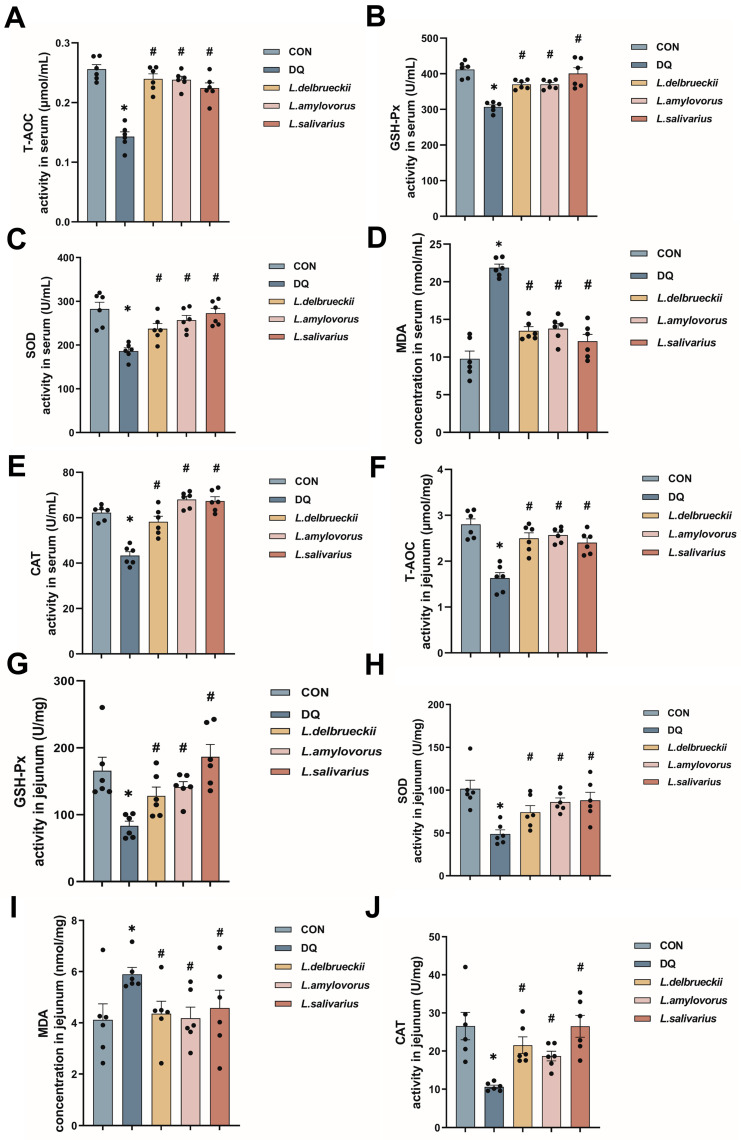
Effect of *Lactobacillus* on oxidation indexes in mice. (**A**–**C**) T-AOC, GSH-Px, and SOD activity in the serum. (**D**) MDA content in the serum. (**E**) CAT activity in the serum. (**F**–**H**) T-AOC, GSH-Px, and SOD activity in the jejunum. (**I**) MDA content in the jejunum. (**J**) CAT activity in the jejunum. Data are presented as mean ± SEM. * *p* < 0.05 when compared to the CON group; # *p* < 0.05 when compared to the diquat group. CON, control; DQ, diquat; *L. delbrueckii*, *Lactobacillus delbrueckii subsp. bulgaricus*; *L. amylovorus*, *Lactobacillus amylovorus*; *L. salivarius*, *Ligilactobacillus salivarius*.

Analysis revealed that T-AOC, GSH-Px, SOD, and CAT activity levels in the diquat group were significantly lower than in the CON group, and MDA content was significantly higher. In contrast, the serum T-AOC, GSH-Px, SOD, and CAT activities in the *L. delbrueckii*, *L. amylovorus,* and *L. salivarius* groups were significantly higher than in the diquat group, and the MDA level was significantly lower (Figure 2A–E; *p* < 0.05). Similar differences between the groups were also noted in the jejunum, except for the similar MDA content in the *L. salivarius* and diquat groups. These results suggested that *L. delbrueckii*, *L. amylovorus*, and *L. salivarius* significantly alleviated the oxidative stress caused by diquat (Figure 2F–J; *p* < 0.05).

### 3.3. Lactobacillus Can Regulate the Disturbance Induced by Diquat to the Gut Microbiota

**Figure 3:**
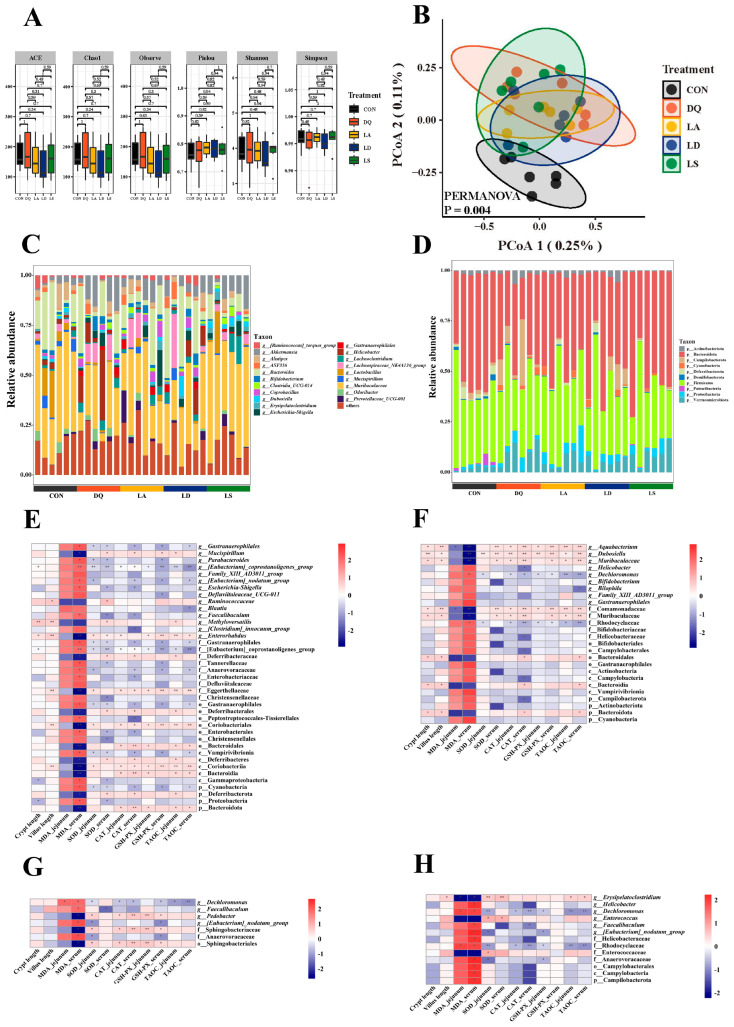
Effect of *Lactobacillus* on the composition of the jejunal microorganisms. (**A**) Alpha diversity. (**B**) Beta diversity. (**C**) Histogram of species composition by genus. (**D**) Histogram of species composition by phylum. (**E**) Heat map of the correlation between the CON and diquat groups. (**F**) Heat map of the correlation between *L. salivarius* and the microorganisms in the diquat group. (**G**) Heat map of the association between *L. delbrueckii* and the microorganisms in the diquat group. (**H**) Heat map of the association between *L. amylovorus* and the microorganisms in the diquat group. CON, control; DQ, diquat; *L. delbrueckii*, *Lactobacillus delbrueckii subsp. bulgaricus*; *L. amylovorus*, *Lactobacillus amylovorus*; *L. salivarius*, *Ligilactobacillus salivarius*. * *p* < 0.05; ** *p* < 0.01.

Alpha diversity analysis showed similarity among the groups (Figure 3A). PCoA showed that the CON group was separated from the other groups, suggesting significant differences in the abundance of their gut microbial species (Figure 3B). At the phylum level, the jejunal microbiota was mainly composed of Bacteroidetes, Firmicutes, and Proteobacteria; at the genus level, *Muribaculaceae*, *Bacteroides*, and *Akkermansia* were the dominant genera. In addition, compared to the CON group, the diquat group showed a significant decrease in the abundance of Bacteroidota, Coriobacteriia, and *Enterorhabdus*; a significant increase in the abundance of *Escherichia–Shigella* and [*Eubacterium*] *coprostanoligenes groups* (Figure 3C,D and Figure S1).

Spearman’s correlation analysis further confirmed the correlation between gut microbiota and oxidative stress indicators. Through analysis of the bacteria in the diquat and CON groups, we found that the [*Euprostanoligenes*] *coprostanoligenes* group was negatively correlated with the activity of SOD, CAT, T-AOC, and GSH-Px and positively correlated with MDA content. Coriobacteriia, Eggerthellaceae, and Bacteroidota were positively correlated with SOD, CAT, T-AOC, and GSH-Px activity, and negatively correlated with MDA content.

The abundance of *Dechloromonas* in the three *Lactobacillus* groups was significantly lower than in the diquat group and was negatively correlated with the activities of SOD, CAT, T-AOC, and GSH-Px and positively correlated with MDA content, suggesting that the three *Lactobacillus* species inhibited the oxidative stress damage caused by diquat by regulating *Dechloromonas* (Figure 3E–H).

### 3.4. EV Treatment Ameliorates Diquat-Induced Weight Loss and Jejunal Damage

**Figure 4:**
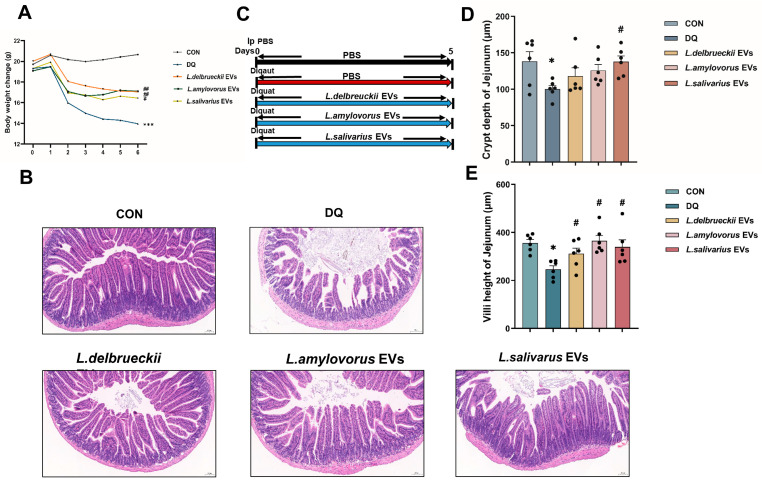
Effect of *Lactobacillus* EVs on weight loss and oxidative damage caused by diquat. (**A**) Mice weight change (*n* = 6). (**B**) H&E staining of the jejunum. (**C**) Experimental grouping scheme. (**D**) Jejunal crypt depth. (**E**) Jejunal villi height. Data are presented as mean ± SEM. * *p* < 0.05 and *** *p* < 0.001 when compared to the CON group; # *p* < 0.05 and ## *p* < 0.01 when compared to the diquat group. PBS, phosphate-buffered saline; CON, control; DQ, diquat; *L. delbrueckii*, *Lactobacillus delbrueckii subsp. bulgaricus*; *L. amylovorus*, *Lactobacillus amylovorus*; *L. salivarius*, *Ligilactobacillus salivarius*.

The three *Lactobacillus* EVs significantly alleviated diquat-induced body weight loss in mice, suggesting they played a positive role (Figure 4A). H&E staining showed that the EVs alleviated the intestinal epithelial damage caused by diquat, mainly by increasing the number, density, and neat arrangement of small intestinal microvilli, similar to the effects delivered by the administration of each of the three *Lactobacillus* species alone (Figure 4E; *p* < 0.05). The jejunal villi in the *L. delbrueckii*, *L. amylovorus*, and *L. salivarius* EV groups were significantly higher (Figure 4C; *p* < 0.05) and the jejunal crypts in the *L. salivarius* EV group were significantly deeper (Figure 4D; *p* < 0.05) than in the diquat group. These findings highlight the difference in impact between the EVs and each of the three *Lactobacillus* species alone.

### 3.5. EV Treatment Reduced Oxidation Indicator Levels in the Serum and Jejunum

**Figure 5:**
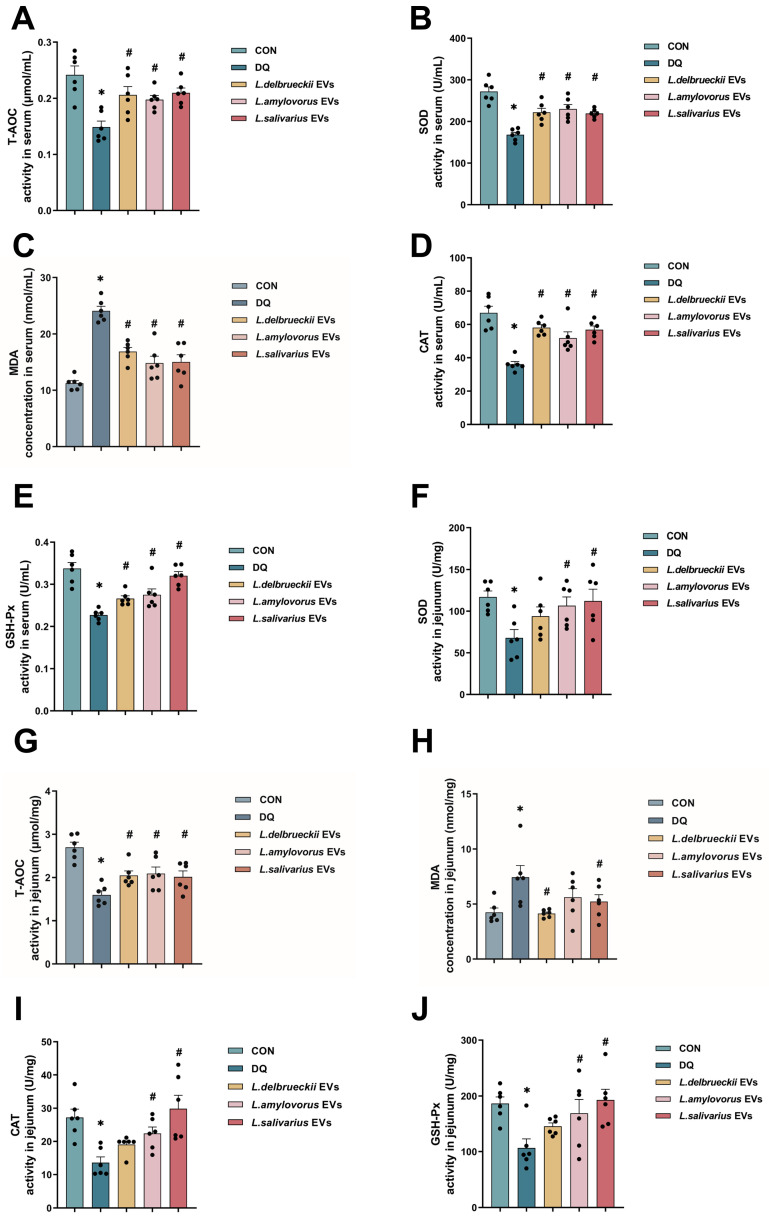
Effect of *Lactobacillus* EVs on oxidation indicators in mice. (**A**,**B**) T-AOC and GSH-Px activity in the serum. (**C**) MDA content in the serum. (**D**,**E**) SOD and CAT activity in the serum. (**F**,**G**) T-AOC and GSH-Px activity in the jejunum. (**H**) MDA content in the jejunum. (**I**,**J**) SOD and CAT activity in the jejunum. Data are presented as mean ± SEM. * *p* < 0.05 when compared to the CON group, # *p* < 0.05 when compared to the diquat group. CON, control; DQ, diquat; *L. delbrueckii*, *Lactobacillus delbrueckii subsp. bulgaricus*; *L. amylovorus*, *Lactobacillus amylovorus*; *L. salivarius*, *Ligilactobacillus salivarius*.

The oxidative stress indicators in the serum revealed that the *Lactobacillus* EVs had the same effect as the direct intervention with *Lactobacillus*. The *L. delbrueckii*, *L. amylovorus*, and *L. salivarius* EV groups had higher serum T-AOC, GSH-Px, SOD, and CAT activities and lower MDA levels than the diquat group (Figure 5A–E; *p* < 0.05).

The oxidative stress indicators in the jejunum showed slight differences between the effects of the *Lactobacillus* EVs and the direct intervention with *Lactobacillus*. The *L. delbrueckii*, *L. amylovorus*, and *L. salivarius* EV groups had significantly higher T-AOC activity than the diquat group. The *L. amylovorus* and *L. salivarius* EV groups had significantly higher SOD, CAT, and GSH-Px activities than the diquat group, while their activities in the *L. delbrueckii* EV and diquat groups were similar. The *L. delbrueckii* and *L. salivarius* EV groups had significantly lower MDA levels than the diquat group, while the *L. amylovorus* EV and diquat groups were similar (Figure 5F–J; *p* < 0.05).

### 3.6. EV Therapy Modulated Diquat-Induced Disordered Gut Microbiota

**Figure 6:**
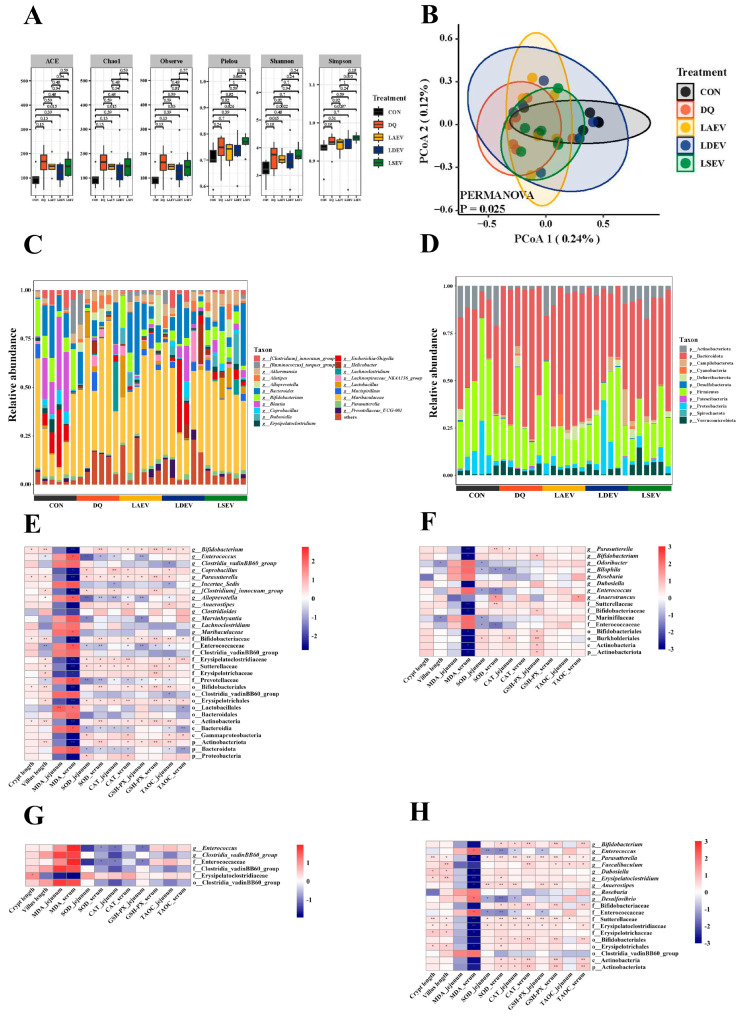
Effect of *Lactobacillus* EVs on the composition of the jejunal microorganisms. (**A**) Alpha diversity. (**B**) Beta diversity. (**C**) Histogram of species composition by genus. (**D**) Histogram of species composition by phylum. (**E**) Heat map of the correlation between the CON and diquat groups. (**F**) Heat map of the correlation between the *L. amylovorus* EV and the microbial composition in the diquat groups. (**G**) Heat map of the association between *L. delbrueckii* EVs and the microbial composition in the diquat group. (**H**) Heat map of the association between *L. salivarius* EVs and the microbial composition in the diquat group. CON, control; DQ, diquat; *L. delbrueckii*, *Lactobacillus delbrueckii subsp. bulgaricus*; *L. amylovorus*, *Lactobacillus amylovorus*; *L. salivarius*, *Ligilactobacillus salivarius*. * *p* < 0.05; ** *p* < 0.01.

Alpha diversity showed that probiotic EVs increased microbial diversity (Figure 6A). PCoA showed insignificant separation of the intestinal microbial community structure in each group, which differed from the interventions that used *Lactobacillus* alone (Figure 6B). Analysis of the gut microorganism composition revealed that Firmicutes, Bacteroidota, Verrucomicrobiota, and Proteobacteria were the dominant organisms at the phylum level, while *Bacteroides* were the dominant organisms at the genus level (Figure 6C,D).

The abundance of *Bifidobacterium*, *Parasutterella,* and Erysipelatoclostridiaceae in the diquat group was significantly lower than in the CON group. *Enterococcus* in the EV groups was significantly lower and the abundance of *Bifidobacterium* and *Erysipelatoclostridium* was significantly higher than in the diquat group (Figure S2).

Spearman’s correlation analysis showed that *Erysipelotrichales* and *Parasutterella* were positively correlated with SOD, CAT, T-AOC, and GSH-Px activities and negatively correlated with MDA content. Bacteroidota were negatively correlated with SOD, CAT, T-AOC, and GSH-Px activities and positively correlated with MDA content. *Enterococcus* was negatively correlated with SOD, CAT, and GSH-Px activities and positively correlated with MDA content (Figure 6E–H).

## 4. Discussion

*Poria cocos*, a dried sclerotia of porous fungi with a brown outer epidermis and a white inner core, has long been used in traditional Asian medicine and has a history of nearly 1000 years as a food. *Poria cocos* is believed to be good at regulating gastrointestinal functions [19]. In recent years, researchers aiming to elucidate the pharmacodynamic mechanism of *Poria cocos* turned their attention to the PCPs which account for 70–90% of its total weight and are its most important bioactive substance [20]. PCPs exhibit beneficial lipid regulation, anti-inflammatory, antioxidant, and immunomodulatory effects [21,22]. Several recent studies have shown that PCPs can alter the gut microbiome to improve normal intestinal physiological function; however, how the altered gut microbiome is involved in improving intestinal physiological function deserves more in-depth study [23,24]. According to our previous research data, feeding PCPs can significantly improve growth indexes and the antioxidant capacity of newborn piglets and increase the abundance of *Lactobacillus* in their intestinal microbiota. Various *Lactobacillus* species were shown to endow multiple beneficial effects [25,26]. *Lactobacillus* can benefit the host by directly acting on the gut microorganisms. These bacteria play a role in competitive adhesion, disruption of the information transmission of harmful microorganisms, and maintenance of the intestinal microecology through their metabolites [27,28]. Furthermore, *Lactobacillus* can directly access the intestinal epithelium and participate in the cell surface factors recognition that modulates epithelial cell anti-inflammatory and anti-apoptotic functions and maintains intestinal epithelial cell viability [29,30,31]. Moreover, *Lactobacillus* can regulate intestinal microecology by controlling specific functions of the mucosal immune system through the mucosal immune system or epithelial cells [32,33]. All these suggest that *Lactobacillus* can significantly improve intestinal antioxidant activity. To test this hypothesis, we conducted the present study using *L. delbrueckii*, *L. amylovorus*, *L. salivarius*, *L. acidophilus*, *L. fermentum*, and *L. animalis* isolated from the intestines of neonatal piglets fed PCPs. Diquat (oxidative stress inducer) was used to establish an intestinal oxidative stress injury model. We aimed to verify the alleviating effect and action mode of the various lactic acid-producing bacteria on oxidative stress injury.

Diquat is an oxidized reduced bipyridine herbicide. It is a moderately toxic compound that uses molecular oxygen to generate oxygen radicals and other reactive oxygen species after entering the animal body, leading to oxidative stress [34,35]. The oxidative stress caused by diquat can impair the normal growth, metabolism, and function of intestinal epithelial cells and trigger an inflammatory response. The oxidative stress also results in extensive intestinal cell apoptosis, damaging the intestinal mucosal morphology, increasing intestinal permeability, and hampering the intestinal immune function, ultimately resulting in a significant loss of body weight [36]. In this experiment, it was found that gavage of *L. delbrueckii*, *L. amylovorus,* and *L. salivarius* significantly alleviated the weight loss caused by diquat-induced oxidative stress (Figure 1C), whereas gavage of *L. acidophilus*, *L. fermentum*, and *L. animalis* did not. Therefore, *L. delbrueckii*, *L. amylovorus,* and *L. salivarius* were selected for subsequent experiments.

H&E staining showed that diquat significantly decreased the crypt depth and villus height of the jejunum (Figure 1C), whereas *L. delbrueckii*, *L. amylovorus*, and *L. salivarius* prevented these changes in the crypt depth, and *L. amylovorus* and *L. salivarius* also in the villus height (Figure 1D,E). Assessment of the oxidative stress indicators showed that *L. delbrueckii* and *L. amylovorus* significantly elevated the activities of T-AOC, GSH-Px, SOD, and CAT and reduced the level of MDA in the serum and jejunum, whereas *L. salivarius* did not affect the jejunal MDA level but was otherwise similar to the other two bacteria. The results of 16S rRNA sequencing showed a significant decrease in the abundance of Bacteroidota, Coriobacteriia, and *Enterorhabdus* in the diquat group and a significant increase in the abundance of *Escherichia–Shigella* and [*Eubacterium*] *coprostanoligenes* (Figure 3C,D and Figure S1). Bacteroidota, Coriobacteriia, and *Enterorhabdus* showed a contributory role in alleviating oxidative stress and maintaining the intestinal barrier, whereas the increase in *Escherichia–Shigella* and [*Eubacterium*] *coprostanoligenes* was closely related to oxidative stress and inflammatory damage, illustrating the contribution diquat made to intestinal oxidative stress damage by remodeling the gut microbiota [37,38,39,40]. *Dechloromonas* abundance was significantly decreased in the *L. delbrueckii*, *L. amylovorus*, and *L. salivarius* groups, and was negatively correlated with SOD, CAT, T-AOC, and GSH-Px activities and positively correlated with MDA content. These findings suggested that *Dechloromonas* abundance was closely related to the oxidative stress status [41]. Therefore, we suggest that *L. delbrueckii*, *L. amylovorus*, and *L. salivarius* inhibit the oxidative stress damage induced by diquat through the regulation of *Dechloromonas* (Figure 3E–H). However, the exact pathway through which this occurs needs to be further investigated.

After verifying that the three *Lactobacillus* species possess antioxidant effects, we attempted to elucidate how they achieve these effects. The bidirectional microbiota–host communication in the gut ecosystem does not involve direct cell contact. Both microorganism- and host-derived EVs are key players in such interkingdom crosstalk. An accumulating body of evidence indicates that bacterial-secreted vesicles mediate the microorganism functions by transporting and delivering effector molecules into host cells to modulate the host’s signaling pathways and cell processes. Consequently, vesicles released by the gut microorganisms could greatly influence the host’s health and disease [42,43]. The host- and microorganism-derived EVs regulate the epithelial barrier integrity, which is critical to gut homeostasis. Disruption of this barrier could lead to increased intestinal permeability, causing inflammatory, oxidative stress, and metabolic diseases [44]. Most recent studies on EVs have focused on Gram-negative bacteria, with just a few conducted on Gram-positive bacteria. Therefore, we extracted the EVs of *L. delbrueckii*, *L. amylovorus*, and *L. salivarius* to investigate their antioxidant effects and explore the link between these and oxidative stress. The results showed that *L. delbrueckii*, *L. amylovorus,* and *L. salivarius* EVs significantly alleviated the weight loss caused by diquat. Unlike the direct infusion of *Lactobacillus*, all three EVs significantly increased jejunal villus height and those of *L. salivarius* also increased the jejunal crypt depth (Figure 4D,E). Serum antioxidant indicator assessment showed that these EVs significantly decreased the MDA content and increased the activity of T-AOC, SOD, CAT, and GSH-Px (Figure 5), in agreement with the direct *Lactobacillus* infusion results. Differences among the three EV groups were noted mainly in the oxidative stress indicators in the jejunum. *L. salivarius* EVs significantly decreased the jejunal MDA content and increased the activity of T-AOC, SOD, CAT, and GSH-Px. The MDA content in the *L. amylovorus* EV group was similar to that of the diquat group. The SOD, CAT, and GSH-Px activity levels in the *L. delbrueckii* EV group were similar to those in the diquat group, while the MDA level in the *L. delbrueckii* EV group was significantly lower. It is well known that T-AOC, SOD, CAT, and GSH-Px are closely related to antioxidant activity, whereas MDA represents the degree of oxidative stress damage. Therefore, our findings suggest that *L. delbrueckii*, *L. amylovorus*, and *L. salivarius* EVs could alleviate the intestinal oxidative stress damage induced by diquat. *L. salivarius* EVs enhanced the jejunal antioxidant enzyme activity and reduced the oxidative damage, *L. amylovorus* EVs enhanced the antioxidant activity but did not affect the level of oxidative damage, and *L. delbrueckii* EVs reduced oxidative damage but did not enhance the antioxidant activity to support the oxidative stress alleviation effect.

The 16S rRNA sequencing results showed a significant decrease in the abundance of *Enterococcus* in the EV groups and a significant increase in the abundance of *Parasutterella*, *Bifidobacterium*, and *Erysipelatoclostridium* (Figure S2). These bacteria are closely related to oxidative stress regulation. For example, the virulence factors secreted by *Helicobacter* activate the oxidative stress signaling pathway and mediate the chronic inflammatory response in the host cells [45]. *Lactobacillus* could enhance the antioxidant capacity by significantly inhibiting *Faecalibaculum* and *Enterococcus* through the Nrf-2 pathway [46]. Spearman’s correlation analysis showed similar findings; Erysipelotrichales and *Parasutterella* were positively correlated with antioxidant enzyme activities and negatively correlated with oxidative damage, and the opposite was true for Bacteroidales and *Enterococcus* (Figure 6E–H). These results suggested that *L. delbrueckii*, *L. amylovorus*, and *L. salivarius* EVs regulated oxidative stress by remodeling the gut microbiota, e.g., by increasing the abundance of Erysipelotrichales and *Parasutterella* and decreasing the abundance of Bacteroidales and *Enterococcus* to mitigate oxidative stress damage.

## 5. Conclusions

*L. delbrueckii*, *L. amylovorus*, and *L. salivarius* EVs remodeled the gut microbiota by decreasing the abundance of harmful bacteria such as *Enterococcus* and increasing that of beneficial bacteria such as *Parasutterella*, *Bifidobacterium*, and *Erysipelatoclostridium*. In turn, these changes improved the intestinal physical barriers, as indicated by the longer intestinal villi and deeper crypts, enhanced the antioxidant enzyme activity, and mitigated the oxidative stress damage caused by diquat. Unlike probiotics that require pre-colonization, EVs can be efficiently applied as they can act directly on the intestinal tract. The experimental results also provided new ideas for developing products to protect intestinal health. However, the activity differences among the *Lactobacillus* EVs and the need for a more comprehensive link between them and the intestinal barrier warrant further investigation.

## Data Availability

Not applicable.

## Supplementary Materials

The following supporting information can be downloaded at: https://www.mdpi.com/article/10.3390/nu15194198/s1, Figure S1: Heat map of microbial species differences after *Lactobacillus* gavage; Figure S2: Heat map of microbial species differences after *Lactobacillus* EV gavage.
