# Supplementary figures and images for: Three Strains of Lactobacillus Derived from Piglets Alleviated Intestinal Oxidative Stress Induced by Diquat through Extracellular Vesicles
Source: Nutrients. 2023 Sep 28;15(19):4198. doi: 10.3390/nu15194198 (PMC10574712; doi:10.3390/nu15194198)

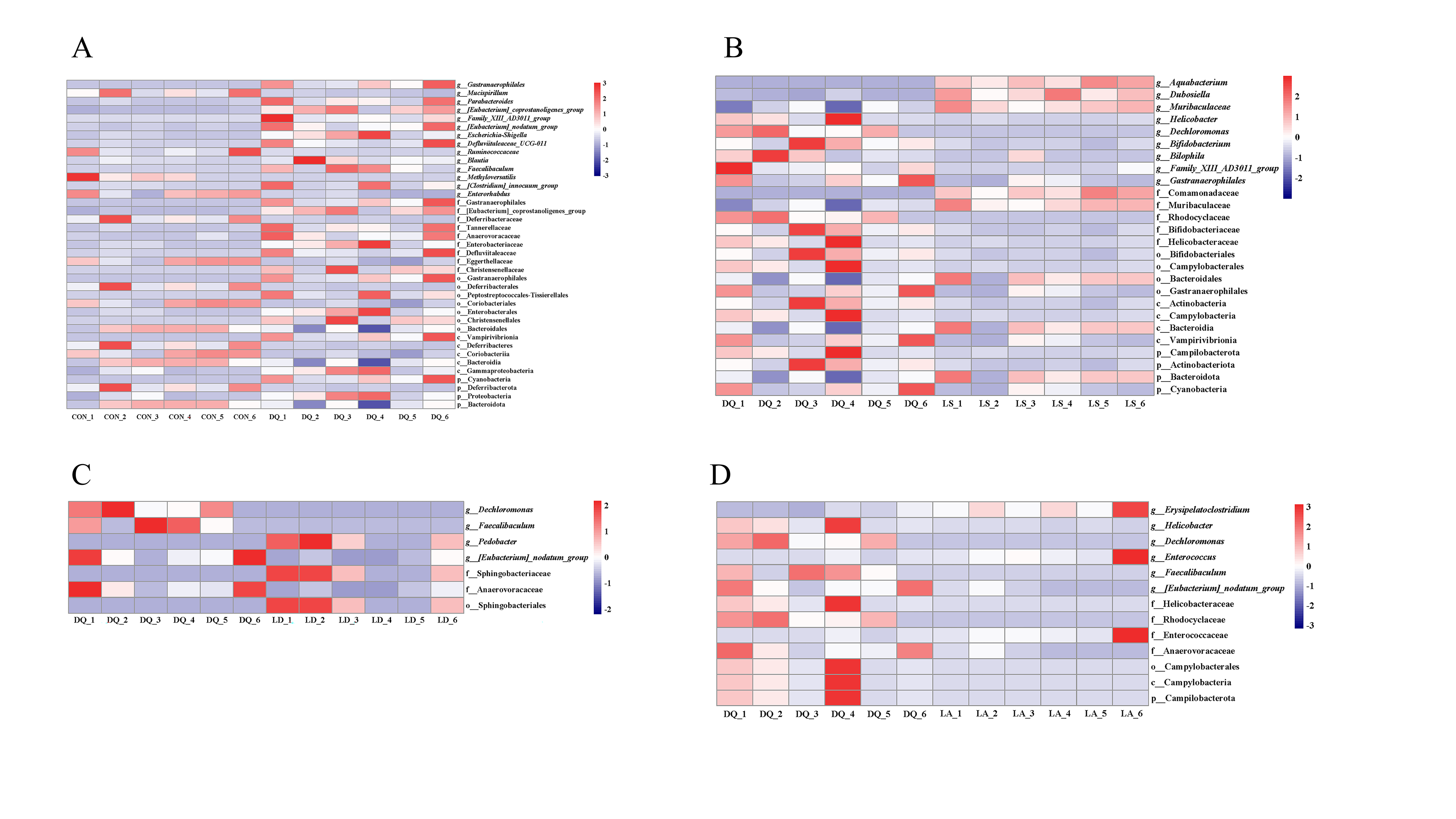

Supplement: Supplementary file 1 [file nutrients-15-04198-s001.zip › Figure S1.tif]

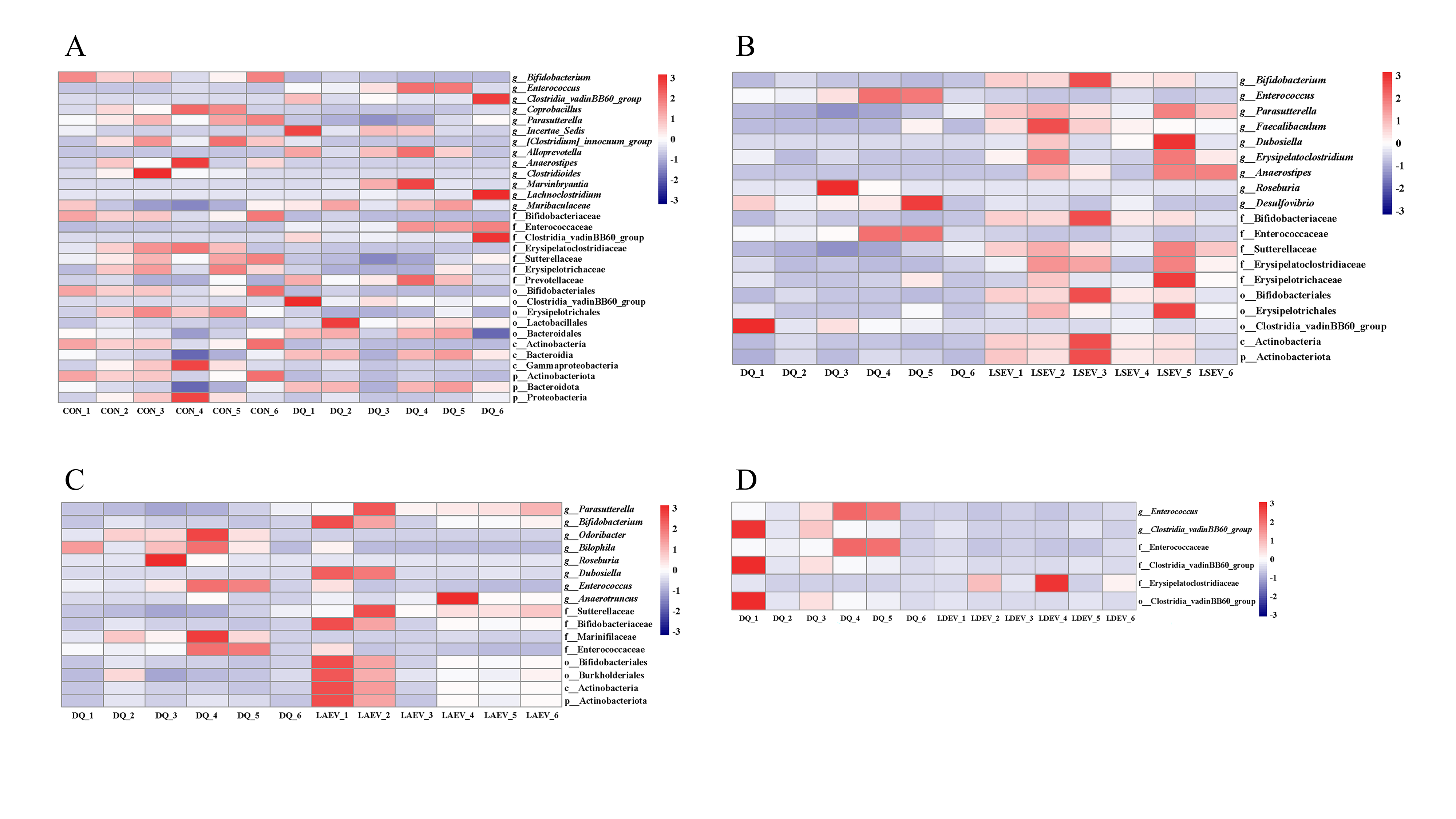

Supplement: Supplementary file 1 [file nutrients-15-04198-s001.zip › Figure S2.tif]
